# High-Risk Neuroblastoma Challenges and Opportunities for Antibody-Based Cellular Immunotherapy

**DOI:** 10.3390/jcm13164765

**Published:** 2024-08-13

**Authors:** Natasha V. Persaud, Jeong A. Park, Nai Kong V. Cheung

**Affiliations:** 1 Department of Pediatrics Memorial Sloan Kettering Cancer Center, New York, NY 10065, USA; persaun1@mskcc.org; 2 Pediatrics Inha University Hospital, Icheon 22332, Republic of Korea; jeonga95@gmail.com

**Keywords:** high-risk neuroblastoma, adoptive cellular therapy, ex vivo armed T cell with bispecific antibody (EAT)

## Abstract

Immunotherapy has emerged as an attractive option for patients with relapsed or refractory high-risk neuroblastoma (HRNB). Neuroblastoma (NB), a sympathetic nervous system cancer arising from an embryonic neural crest cell, is heterogeneous clinically, with outcomes ranging from an isolated abdominal mass that spontaneously regresses to a widely metastatic disease with cure rates of about 50% despite intensive multimodal treatment. Risk group stratification and stage-adapted therapy to achieve cure with minimal toxicities have accomplished major milestones. Targeted immunotherapeutic approaches including monoclonal antibodies, vaccines, adoptive cellular therapies, their combinations, and their integration into standard of care are attractive therapeutic options, although curative challenges and toxicity concerns remain. In this review, we provide an overview of immune approaches to NB and the tumor microenvironment (TME) within the clinical translational framework. We propose a novel T cell-based therapeutic approach that leverages the unique properties of tumor surface antigens such as ganglioside GD2, incorporating specific monoclonal antibodies and recent advancements in adoptive cell therapy.

## 1. Introduction

Hailed as the breakthrough of the decade, cancer immunotherapy has transformed the therapeutic landscape and patient prognosis for both adult and pediatric cancers [1]. Despite the phenomenal successes of immune checkpoint blockades (ICBs) in several adult cancers and chimeric antigen receptor T cells (CARTs) in both adult and pediatric hematological malignancies, the promise of immunotherapy in pediatric solid tumors characterized by extremely low tumor burden (TMB) and scant tumor-infiltrating lymphocytes (TILs) remains elusive [2,3,4,5,6,7].

Neuroblastoma (NB), the most common extracranial solid malignancy in children, predominantly affects young children. Approximately 40% of diagnoses are made within the first year of life, and it accounts for 10–15% of pediatric cancer deaths [8,9]. It is an embryonal tumor derived from differentiation arrest of the neural crest along sympathoadrenal development, exhibiting a heterogeneous clinical presentation and outcome [10]. Half of such tumors present with diffuse metastases, while the rest are a benign solitary mass, with limited local spread, either spontaneously regressing or maturing, or surgically curable. Risk-based therapy to minimize toxicities uses age of patient at diagnosis, extent of disease, tumor histology, MYCN status and DNA index/ploidy as classifiers for treatment recommendations that range from none or minimal for children with low-risk (LR) disease to intensive, multimodal therapy including cytotoxic agents, surgical resection, radiotherapy, immunotherapy, differentiation agents and stem cell transplantation for high-risk (HR) disease [9,10,11,12,13]. In general, low-risk (LR) and intermediate-risk (IR) disease have a favorable prognosis with overall survival (OS) rates of >95%, whereas high-risk (HR) disease, despite intensive multimodal therapy, has a dismal outcome with 5-year survival rates of less than 50% [9]. The underlying genetics of these disparate outcomes have been extensively studied, but the immunological basis is far less complete [14,15].

HRNB is often classified as a “cold” tumor due to the scarcity of tumor-infiltrating lymphocytes (TILs), its poor immunogenicity characterized by low or absent surface expression of major histocompatibility complex class I (MHC class I), and a highly suppressive tumor microenvironment (TME), often associated with defective tumor surveillance or rejection [16]. Classic adaptive immunity refers to de novo T cell immunity, the mastermind of tissue rejection; it is a prerequisite for a response to ICB therapy, which is so far lacking in HRNB. In the absence of T cell responses, passive immunotherapy using monoclonal antibodies (mAb) and adoptive cellular therapies (ACT) has been the mainstream of current therapeutic approaches.

## 2. Biological Features of Neuroblastoma in Risk Assignment and Treatment

The clinical heterogeneity of NB underscores the complex biology of normal development and abnormal tumorigenesis along the sympathoadrenal lineage. The differentiation and maturation pathways of the neural crest cells are tightly regulated by numerous factors that control signaling pathways, cellular interactions, and cellular trafficking. Somatic mutations perturbing transcription and epigenetic dysregulation during the developmental process block differentiation of neural crest cells and promote tumorigenesis [17]. For example, while transient low expression of MYCN promotes neural crest cell migration and neuronal cell fate specification [18] persistent high MYCN expression leads to sympathoblast hyperplasia and NB formation [10,19].

NB with a more differentiated phenotype is described as favorable histology (FH), while those with less differentiated and primitive characteristics are labeled as unfavorable histology (UFH) according to the International Neuroblastoma Pathology Classification (INPC). Spontaneous remission or differentiation/maturation into ganglioneuroma is common in infants with FH NB, which shows abundance of Schwannian cell stroma and is accompanied by hyperdiploidy and whole chromosomal gains. On the other hand, UFH is more prevalent in older children with more complex and heterogeneous genetics, including *MYCN* amplification (MYCN-A) and/or segmental chromosomal gains or losses (1p deletion, 11q deletion and 17q gain), along with other somatic aberrations such as telomerase reverse transcriptase (*TERT*) rearrangement, and *ATRX* and *ALK* mutations [20,21,22,23]. Despite the low tumor mutational burden (TMB) and few recurrent drivers of tumorigenesis, mutations affecting the telomere maintenance mechanism (TMM), including *MYCN-A*, *TERT-RA* and *ATRX* appear to be the major oncogenic drivers in HRNB [24]. *MYCN*, an oncogene when amplified, remains one of the strongest predictors of high-risk (HR), often associated with a more aggressive disease and shorter progression-free survival (PFS) [25,26]. Newly diagnosed NB with *MYCN-A* is classified as HR regardless of other clinical or biological features and treated aggressively from the time of diagnosis. *TERT* is a direct transcriptional target of the MYCN family of proteins. Both *MYCN* amplification and *TERT* rearrangement (*TERT-RA*) are associated with very poor prognosis in NB [21,23]. *TERT* and *ATRX* mutations are key driver mutations in HR disease that can be acquired during disease progression [15]. Anaplastic lymphoma kinase (*ALK*), another common mutation found in NB, although not associated with HR disease in the absence of *MYCN-A*, is often mutated during disease progression and can be targeted by ALK inhibitors [27].

In addition to genetic mutations, epigenetic factors also contribute to disease pathogenesis and progression. Transcriptional and epigenetic profiling of NB cells has identified two distinct tumor cell types, mesenchymal (MES) and adrenergic (ADRN) types, which differ in transcriptomic, phenotypic, and super-enhancer (SE) expressions and might influence clinical outcomes [28]. This lineage plasticity was observed decades ago by Biedler et al. [29,30] under the microscope, where NB cells evolve along distinct morphologies, neuroblastic (N-type) versus the substrate adherent (S-type), with vastly different growth kinetics and immune profiles [31], prescient of ADRN-MES lineages in surgical samples. The different responses of MES-NB and ADRN-NB to chemotherapy, immunotherapy and targeted therapy coincide with early observations on the N and S cell types. Taken together, clinical heterogeneity of HRNB may arise from NB cells’ attempts to endure ongoing DNA damage and stress. This survival strategy involves upregulating TMM, which in turn controls cell fate [28]. The persistent DNA damage is indicated by the presence of mutational single-base substitution 18 (SBS18) signatures in the majority of HRNB cases. These signatures not only signify the genomic scars from oxidative damage since the tumor’s inception but also indicate ongoing genomic evolution aimed at repairing telomere DNA damage [32]. Normally the body should sense such DNA aberrations and mount an immune response, but NB is able to sabotage any trace of adaptive or innate immunity initiated by the patient, probably through reshaping the TME.

## 3. Immunological Features and Tumor Microenvironment in Neuroblastoma (Table 1)

The inability to eliminate “developmental or embryonal tumor” can be blamed partly on the immaturity of the immune system which is not fully trained to kill non-self and/or abnormal self. The dysregulated oncofetal antigens, regarded as self-antigens, bind to their cognate T cell receptors (TCR) with high affinity, leading to depletion of these T cells during thymic education. As a result, tumor-associated-antigen (TAA)-specific T cells are scarce in the peripheral blood or among TILs in NB. Transcriptional downregulation of MHC class I and defective antigen processing in NB sabotage neoepitopes from stimulating a successful adaptive immune response [25]. The low levels of tumoral MHC I expression can also be attributed to undifferentiated neural crest development [10]. Natural killer (NK) cell-mediated cytotoxicity against the “abnormal self” is also compromised because of the low levels of NK cell-activating molecules in NB [33,34]. Direct tumor cell phagocytosis by macrophages can also be compromised via the surface expression of “do-not-eat-me” checkpoint molecule CD47 plus GD2 interaction with the inhibitory immunoreceptor Siglec-7 in NB [35]. In addition, an abundance of immunosuppressive molecules, including B7-H3 and cytokines such as IL-10 and TGFβ, further intensify the suppressive milieu of the TME [25] Bernards 1986. Furthermore, tumor infiltrating immunosuppressive cells, including tumor-associated macrophages (TAMs), myeloid-derived suppressor cells (MDSCs) and regulatory T cells (Tregs), cooperate to induce apoptosis of cytotoxic T lymphocytes (CTL) and T helper type 1 (Th1) cells, thereby de-railing the initiation and the execution of active immune responses [36]. Because of this immunosuppressive TME, HRNB is typically “cold”, with few or no TILs [37], which is best highlighted among MYCN-A tumors (17) [16]. In contrast, among low-risk (LR) and intermediate-risk (IR) tumors associated with better progression-free survival (PFS) and overall survival (OS) [38], TME shows an abundance of professional CTL and Th1 cells. The multitude of hurdles for a meaningful T cell immunity not only precludes effective tumor surveillance in HRNB, it also nullifies strategies to activate host-derived T cell immunity through immune activators or checkpoint inhibitors [14].

The heterogenous population of immunosuppressive cells not only contributes to immune suppression, but also to tumor angiogenesis and tumor cell metastases [39,40,41]. T cell metabolism, homing and activation are perturbed by a pletora of mechanisms, including key amino acid hoarding [42,43,44], cleaving of L-selectin [45] and upregulation of PD-L1 [46]. These mechanisms are not only relevant for native T cells, but most likely present major hurdles to adoptive T cell-based immunotherapy.

The insatiable need for the acquisition of blood supply for tumor growth and the resulting immature choatic microvasculature further ensures a highly hypoxic and inflammatory TME [47,48,49]. Altogether, native adaptive immune activation for clearance of the “immature self” and/or “abnormal self” fails, and tumor progression and therapeutic resistance becomes inevitable [48,49,50].

Adoptive T-cell immunotherapy platforms that support combinatorial strategies to deliver potent polyclonal T cells while simultaneously targeting the abnormal tumor microvasculature and immunosuppressive cellular and molecular entities of TME should be exploited. The immunologic features of NB and its TME are summarized in Table 1.

**Table 1:** The limitations and opportunities in neuroblastoma and tumor microenvironments. Antibody-mediated adoptive cellular therapy using ex vivo armed T cells (EAT) when combined with small molecule or biologic modifiers could overcome the inhibitory TME in HRNB.

	Limitations	Opportunities
Neuroblastoma (NB)	Low MHC class I expression Low tumor mutational burden (TMB)	Adoptive Cell Therapy. GD2 is a reliable tumor-associated surface antigen, an ideal target for novel antibody-based therapeutics.
Tumor micro-environment (TME)	Immature microvasculature Excess immunosuppressive cells Immunosuppressive pathways Few tumor-infiltrating lymphocytes (TILs)	VEGF inhibitors. Antibody-depletion protocols. Immune checkpoint blockades. Adoptive Cell Therapy using ex vivo expanded TIL, chimeric antigen receptor modified T cells (CARTs) and T cells armed ex vivo with T-BsAb.

## 4. Immunotherapies for Neuroblastoma: Monoclonal Antibody, Chimeric Antigen Receptor T Cells, and Bispecific Antibodies

Despite aggressive multimodal treatments, at least 40% of children with HRNB still succumb to disease [51]. This is the impetus for developing novel treatment strategies such as targeted small molecules or immunotherapies. GD2, a well-established TAA with stable surface expression, has become an attractive target for NB immunotherapy [52]. Anti-GD2 mAb-based immunotherapy has demonstrated its clinical efficacy and safety in several versions, including murine IgG3 3F8 and 14G2a, chimeric ch14.18, and their humanized versions hu3FA and hu14.18-K322A, respectively, engaging NK cells and myeloid effector cells in antibody-dependent cell cytotoxicity (ADCC), while activating complement-dependent cytotoxicity (CDC) [53]. Despite the clinical success of anti-GD2 mAbs, challenges remain both in efficacy and toxicity. Once the importance of T cells was realized with the advent of ICBs, genetic engineers rapidly applied recombinant tools to reshape IgGs to engage T cells to exploit their “professional killer” capabilities. The fields of CARTs and bispecific antibody (BsAb)-engaging T cells rapidly evolved [14,54,55,56]. These passive T cell immunotherapies directed at high density surface antigens can overcome several limitations, e.g., low TMB, paucity of neoantigens, absent/few TILs, and downregulation of MHC class I/defective antigen presentation machinery. While CARTs have been clinically successful in hematologic malignancies, for solid tumors and specifically HRNB, they remain early in development [57] Del Bufalo. Trafficking into solid tumors is limited, and persistence of CARTs remains inadequate due to early exhaustion and depletion from activation-induced cell death (AICD) [58]. Their acute and long-term toxicities, exemplified by cytokine release syndrome (CRS), immune effector cell-associated neurotoxicity syndrome (ICANS), and potentials for secondary malignancies, though manageable, are still life-threatening [59]. In addition, as a personalized medicine, CART manufacturing is elaborate, expensive and so far, affordable only in developed countries [60].

On the other hand, T cell-engaging BsAb exploiting the proven tumor selectivity of anti-GD2 mAb and OKT3 to direct polyclonal CD3(+) T cells into “cold” GD2-positive tumors has achieved tumor ablation without the need for ADCC or CDC [14]. The ability of pan-T cell activation (CD4^+^, CD8^+^, αβ^+^ and γδ^+^ T cells) underlies its potency, eliminating the need for additional co-stimulatory signals (e.g., CD28 or 41BB required for CART) and thereby reducing exhaustion and AICD [58]. Pre-clinical studies comparing GD2-CARTs and T cells stimulated with GD2-BsAb showed that BsAb-stimulated T cells survived for an extended period, while CARTs with high density receptors became easily exhausted and depleted when incubated with tumor cells [58].

## 5. Ex Vivo Armed T Cell with Bispecific Antibodies (EATs)

**Figure 1:**
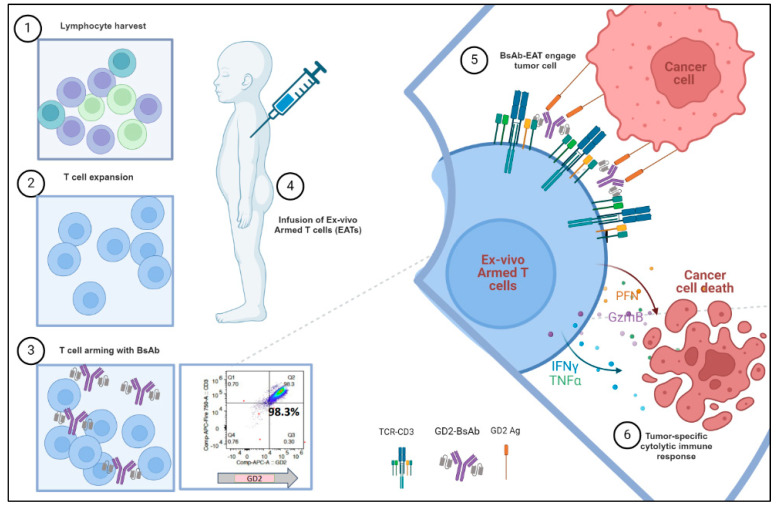
Antibody-based adoptive cellular therapy. Ex vivo armed T cells with BsAb (EATs) are manufactured as follows: (1) lymphocyte harvest, (2) T cell expansion over 10–14 days, (3) T cell arming with BsAb (efficiency of ≥98% versus transduction efficiency of 80% with hu3F8CAR) [58], (4) cryopreservation, and (5) thawing before infusion (without lymphodepletion). EATs home to tumors to engage GD2, whereby they become activated, form synapses with NB, and release cytolytic granules (perforin PFN, granzyme GzmB) plus cytokines (interferon gamma, IFN-ϒ and tumor necrosis factor alpha, TNF-α), effecting tumor kill.

Previously, we demonstrated that ex vivo armed T cells with bispecific antibodies (EATs) effectively traffic to tumor sites, infiltrate the tumor parenchyma, and exhibit potent cytotoxicity against various tumor xenografts, including NB [14]. Instead of gene modification, EATs carry BsAb molecules attached via their surface CD3, and remain quiesent until they home to tumors, where engagement with tumor targets immediately initiates a cascade of downstream signals leading to T cell activation and tumor cytolysis (Figure 1) [14,61].

Unlike the all-in-one design of classic CARTs, where the driver (anti-antigen scFv of CAR) is permanently transduced onto T cells, BsAb is an off-the-shelf agent applicable for any CD3(+) T cells. Similar to CARTs, EATs bypass the classic HLA restriction by virtue of their target class (being surface antigens), but unlike CARTs which rely on an elaborate signaling pathway optimized with an array of co-stimulatory molecules, EATs do not require additional co-stimulatory activation, avoiding the over-activation or early exhaustion of T cells. Unlike CARTs persisting as life-long drugs where toxicity (e.g., CD19(+) B cell aplasia) can be permanent, EATs lose their surface-bound BsAb with time, rendering their toxicities self-limited. In addition, a washing step before infusion removes all the nonspecific cytokines released. A comparison of ACT options for NB is presented in Table 2.

### 5.1. Optimizing Bispecific Antibody Platform for EATs

Previously, several clinical trials of EATs, including breast, prostate, and pancreatic cancer, non-Hodgkin lymphoma, multiple myeloma and NB, have documented remarkable safety with evidence of anti-tumor effect [62,63]. GD2-EATs were safe at a T cell dose of 160 million cells/m^2^ × 8 doses, without dose limiting toxicities, especially without CRS and ICANS [63]. The chemical conjugated form of BsAb was optimized by genetic engineering to produce the IgG-[L]-scFv-platformed BsAb, named the “da Vinci” format because of its depiction similar to the Vitruvian Man of perfect proportions [14,64]. This symmetric, dual bivalent IgG-[L]-scFv platform uses scFvs against human CD3ε (huCD3 ε), replacing immunogenic mouse anti-CD3, which to the C-terminus of each light chain in the IgG, optimized not just for affinity, valency and avidity, but also for spatial restrictions. Structural analysis demonstrated superiority for 2 + 2 valency, cis-configuration and a critical interdomain distance. Specifically, the 2 + 2 format (two tumor-binding domains + two CD3-binding domains) was substantially more potent than a 2 + 1 format (two tumor-binding domains + one CD3-binding domain), and cis-configuration (the placement of both the tumor- and T cell-binding domains on the same side) of the BsAb further enhanced in vitro cytokine release and in vivo cytotoxicities [64]. This IgG-[L]-scFv BsAb exerts potent cytotoxicities against multiple tumor antigens including GD2 [47], CD33 [65], GPA33 [66], HER2 [67] and STEAP1 [68] in both in vitro and in vivo settings.

GD2-EATs armed with the “da Vinci” BsAb demonstrated a robust capacity to kill and release Th1 cytokines upon engagement of GD2(+) tumor targets, but with significantly reduced systemic cytokine release and neurotoxicity, contrasting with GD2-CARTs [61,69]. In addition, as demonstrated in GD2(+) melanoma and NB xenografts, tumor regression was accomplished even with low dose of 0.05 μg BsAb/millions of T cells, potentially further reducing the likelihood of systemic toxicities [70]. (See Table 3 for selected pre-clinical and clinical studies using the BsAb-armed T cells immunotherapy platform).

#### 5.1.1. Targeting the TME with EATs: The IMPROVE Strategy 1

A recent clinical study using GD2-CART in HRNB showed promise [57], although the rest of GD2-CART studies so far have been less encouraging. Rapid expansion of MDSCs and TAMs can emerge after CART infusion, limiting the clinical efficacy of GD2-CARTs [78,79]. Similarly, when GD2-EATs infiltrated NB xenografts, expansion of immunosuppressive tumor-infiltrating myeloid cells (TIMs) followed [14]. The TME, a dynamic cellular milieu that promotes tumor angiogenesis, growth, proliferation, and metastasis, can subdue or delay anti-tumor immunity. By combining with specific therapies targeting the immunosuppressive TME, EATs can become supercharged (summarized in Table 1).

One example of TME modulation is depleting TIMs. TIMs constitute a heterogeneous group of suppressive cells in the TME that impede the anti-tumor immune response of T cells and include myeloid-derived suppressor cells, tumor-associated neutrophils (TANs) and tumor-associated macrophages (TAMs). Combining depleting antibodies for each subgroup of TIMs, together with GD2-EATs, not only reduced TIMs, but also improved the efficacy of GD2-EATs. This combination strategy enhances intra-tumoral EAT cell infiltration by up to 14-fold, which translates into a significant improvement in tumor control and survival of the mice treated [14].

Tregs, another prominent immunosuppressive cellular subset in the TME, can abrogate the efforts of even the most efficient cytotoxic T cells. Ipilimumab, an anti-cytotoxic T-lymphocyte antigen-4 (CTLA-4) antibody, is known to inhibit Treg immunosuppression. A component of EAT manufacture, T cell ex vivo expansion and activation in the presence of ipilimumab enhanced T cell proliferation and anti-tumor activity of BsAb-armed T cells (EATs). Specifically, blocking Treg immunosuppression using ipilimumab in combination with EATs (anti-CD3 x anti-EGFR and anti-CD3 x anti-CD20 BsAbs) enhanced tumor-specific cytotoxicity against cancer cell lines COLO356/FG and Daudi, with the increased secretion of chemokines and cytokines [80].

Furthermore, normalization of tumor microvasculature using vascular endothelial growth factor (VEGF) blockades could improve the efficacy of EAT therapy. VEGF blockade using specific antibodies against VEGF or VEGFR2 increased high endothelial venules (HEVs) in the TME and enhanced both cytotoxic CD8(+) and helper CD4(+) cell traffic into TME by up to 8-fold, resulting in improved anti-tumor effects against NB xenografts. This combination immunotherapy strategy was well tolerated in preclinical models, with no additional toxicity observed [14].

Another evasion strategy perturbing anti-tumor immunity is transforming growth factor-beta (TGFβ)-mediated cytotoxic T cell suppression [81,82]. TGFβ is produced by various cell types including MDSCs, cancer-associated fibroblasts (CAFs), TAMs, and activated T cells, as well as malignant cells themselves. TGFβ enhances tumor progression and metastasis by recruiting and polarizing TAMs, immature myeloid cells, and MDSCs (95, 96) [83,84]. Ironically, heightened TGFβ signaling is seen following robust T cell activity such as the release of TGFβ1 from activated CD4(+) Th1 cells [85,86]. While pro-tumorigenic activity of TGFβ signaling was exerted through effects on CD4(+) Th2 cells, a BsAb, CD4-TGFβ Trap (tumor 4T-Trap), which combines a TGFβR2 ligand trap with an anti-CD4(+) T cell-binding antibody, induced cancer hypoxia and cancer cell death, delaying tumor growth in both the MMTV-PyMT breast and and MC38 colon cancer models [87,88] Targeted blockade of TGFβ signaling in Th cells presents an alternative strategy for modulating the TME, which has been pursued to enhance the efficacy of T cell immunotherapies, including ICBs, CARTs, and EATs [88,89,90] (See Table 3 for selected pre-clinical and clinical studies using BsAb-armed T cells immunotherapy platform and combinatorial strategies).

#### 5.1.2. Targeting Multi-Antigen with EATs: The IMPROVE Strategy 2

Tumor heterogeneity contributing to tumor evolution and cancer progression poses another significant challenge, particularly in the context of relapse. This tumor escape mechanism will derail most antigen-specific T cell immunotherapy approaches. One of the most compelling features of EAT lies in its unparalleled ability to swiftly arm diverse BsAbs against multiple antigens without resorting to elaborate genetic modifications, a notable distinction from CART therapy [14]. This inherent advantage not only enhances the plug-and-play adaptability across a diverse array of targets and cancers, but also addresses directly the heterogeneity issue while overcoming the low antigen density hurdle by using cocktails of BsAbs. These multi-EATs, armed ex vivo with multiple BsAbs targeting different tumor-associated antigens (TAAs), can maintain each BsAb’s potency without added toxicity by arming and washing steps. Furthermore, by targeting tumor antigens and cancer stem cell markers simultaneously, multi-EATs should improve the comprehensive approach to eliminate tumors while preventing disease relapse [14].

## 6. Conclusions

Immunotherapy has finally emerged as a viable modality in pediatric cancer as well. Since it does not require cells in their S-phase for effective cytotoxicity, it does not share the toxicities or resistance mechanisms encountered by chemotherapy or radiotherapy. Yet, the immune landscape is challenging because of the scarcity of tumor targets, low TMB with limited tumor neoantigens, and diminished MHC class I expression. GD2 targeted immunotherapies have emerged as a beacon of hope for patients battling with HRNB. Although not a HRNB driver-associated antigen, GD2 is stable and homogenously expressed on NB cell membranes, even in post-treatment settings. GD2 mAb has been proven safe and efficient as part of the current standard of care. However, GD2 mAb can only mediate NK cell and neutrophil cytotoxicity, with help from CDC, and hence, is insufficient for treating bulky disease. Both CART and BsAb can overcome some of the challenges encountered in passive immunotherapies, such as a paucity of neoantigens, low TMB, few driver mutations, and the low MHC class I/defective antigen processing and presentation capacity. While promising, these are still somewhat elusive as single agents.

By optimizing the BsAb format, EATs have demonstrated the potential to become a more specific and potent immunotherapy for high-risk, treatment-resistant NB. While each CART preparation is customized for individual patients, the EAT platform offers an off-the-shelf, straightforward approach to cytotherapy, potentially reducing complexity and cost. More importantly, as the surface attached BsAb wears off with time, the T cells become disarmed, ensuring that toxicities are self-limited. In addition, without tonic activation through artificial costimulatory inserts, EATs are less prone to exhaustion, excessive cytokine surges, or AICD. The addition of small molecules or mAbs that can modify the TME can greatly enhance EAT infiltration into tumors and their cytotoxic potential.

## 7. Future Directions

Ongoing research to understand the genetic and epigenetic signatures involved in the transition from MES to ADREN phenotype should remain a top priority, as factors favoring the ADREN phenotype are associated with sustained GD2 expression and are key to GD2-guided therapeutics. Improvement in T cell-based approaches and the pharmacokinetics of antibody/drug delivery should lessen side effects and allow dose escalation to achieve cures. By combining antibodies, cells and the proven modalities, the standard of care will continue to evolve and approach the ultimate goal of achieving cures, while preserving body functions, in the upcoming decades for every child with HRNB.

## Figures and Tables

**Table 2:** Comparison of adoptive cellular therapy options for neuroblastoma.

	Tumor Infiltrating Lymphocytes (TILs)	Chimeric Antigen Receptor T Cells (CARTs)	Bispecific Antibody (BsAb) PLUS T Cells	Ex Vivo BsAb-Armed T Cells (EATs)
**Description**	Natural TILs ex vivo expanded	T cells transfected with Chimeric Antigen Receptor	BsAb targets T cells to tumor (BsAb as drug)	T cells coated with BsAb to target tumor (T cell as drug)
Traffic to Tumor	Unknown	Good	Good	Good
Tumor-Associated Antigens (TAAs)	Multiple	Single	Single	Multiple
Dominant Lymphocyte Subset	CD8+	CD8+	CD8+	CD8+ and CD4+
MHC class I dependent	Yes	No	No	No
Additional Co-Stimulator Signals Required	Yes	Yes	No	No
Cytokine Storm	Yes	Yes	Yes	Minimized
Persistence	Life Time	Life Time	<1 month	<1 month
Exhaustion	Yes	Yes	Less	Less
Toxicity	CRS	CRS, ICANS	CRS	CRS
Manufacture (Ease/Cost)	Complex/Expensive	Complex/Expensive	Facile/Affordable	Facile/Affordable
Compatible with Other Biologics and Drugs	n/a	Yes	Yes	Yes
Current Stage	Pre-clinical	Early clinical	Pre-clinical	Pre-clinical

**Table 3:** Selected pre-clinical and clinical studies of the BsAb-armed T cell immunotherapy platform.

Antigen	Cancer Patients/Xenographs/Cell Lines	Significance Finding	Ref
GD2, HER2, EGFR CD20	Breast, prostate, pancreatic, non-Hodgkin lymphoma, multple myeloma	Pre-clinical studies: BsAb-armed T cells showed antigen specific proliferation, cytotoxicity and Th1 cytokine release. Phase I and II studies: BsAb successfully redirected armed T cells for non-MHC restricted cytotoxicity for anti-tumor immune response in several malignancies.	[55,71,72,73,74,75]
GD2	Neuroblastoma, osteosarcoma	Phase I study. GD2 BsAb-armed T cells stimulated endogenous T and NK cells for a safe and effective anti-GD2 positive immune response against GD2 positive pediatric malignancies.	[76]
GD2 CD33	Melanoma, neuroblastoma	Bs Ab Platform optimization: 2 + 2, interdomain distance, cis-trans configuration, affinity, valency, avidity, and heterodimeric optimized forms improved cytotoxicity (up to 2000 fold) with enhanced cytokine release and in vivo anti-tumor responses.	[64]
STEAP1	Ewing sarcoma, prostate cancer and canine osteosarcoma cell lines	Superior format over other antibody formats: 2 + 2 IgG-[L]-scFc platform increased TILs into tumor (30 fold) for a superior anti-tumor effects as BsAb alone or ex vivo armed T cells (EATs).	[68]
GD2 HER2	Melanoma, osteosarcoma, small cell lung cancer	Reverse immunosuppressive TIMs: 2 + 2 IgG[L]-scFv platform increased TIL as well as tumor immunosuppressive myeloid (TIM) cells into the TME. Combining depleting protocols to reduce TIMs further increased T cell infiltration and persistence in TME for superior tumor control and improved survival.	[14]
GD2, HER2, GPC3	Neuroblastoma and osteosarcoma.	Normalize neovasculature (VEGF and VEGFR2). Vascular endothelial growth factor (VEGF) blockade improved BsAb-driven intratumoral T cell infiltration (2–8 fold), with more CD8 TIL (than CD4) to yield superior anti-tumor effects without added toxicities.	[14]
GD2 HER2	Osteosarcoma	Immune checkpoint inhibitors (PD1 and PD-L1): BsAb alone or T cells armed with anti-GD2 or anti-HER2 BsAb exerted potent anti-tumor effects in vitro and in vivo. Anti-PDL-1 added sequentially and continuously enhanced BsAb-armed T cells function in vivo for improved tumor control and survival.	[14]
GD2 HER 2	Neuroblastoma, osteosarcoma, primitive neuroectodermal tumor, prostate cancer and melanoma	EATs (Ex vivo armed T cells) alternative to CART: EATs using BsAb on IgG-[L]-scFv-platform exerted safe potent anti-tumor activities against a spectrum of human cancer targets.	[61]
GD2 HER2 CD33 STEAP-1 PSMA	Neuroblastoma, osteosarcoma, primitive neuroectodermal tumor cell line, breast cancer	Overcoming tumor heterogeneity with multispecificity BsAb: BsAb built on IgG-[L]-scFv can support mutli-EAT strategies (T cells armed with BsAb specific for two tumor-associated antigens). Multi-EAT strategies can overcome the heterogeneity associated with tumor evolution. Multi-EAT retains and delivers multiple antigen specificity for adequate anti-tumor potency to suppress tumor growth and without clinical toxcities. This approach prevents clonal escape and prevents cancer resistance.	[14]
77EGFR, HER CD33	Pancreatic ductal adenocarcinoma	Heterodimeric T-BsAb (HDTV) avidity and affinity tuning: 2 + 2 Ig-[L]-scFv T- BsAb format with two anti-CD3 scFv engaged T cells with bivalent binding to tumor antigens with both Fab arms demostrated high avidity (and efficient infiltration of polyclonal T cells into TME for potent T-mediated anti-tumor effects.	[77]
